# Deep-Learning Algorithm and Concomitant Biomarker Identification for NSCLC Prediction Using Multi-Omics Data Integration

**DOI:** 10.3390/biom12121839

**Published:** 2022-12-08

**Authors:** Min-Koo Park, Jin-Muk Lim, Jinwoo Jeong, Yeongjae Jang, Ji-Won Lee, Jeong-Chan Lee, Hyungyu Kim, Euiyul Koh, Sung-Joo Hwang, Hong-Gee Kim, Keun-Cheol Kim

**Affiliations:** 1 Department of Biological Sciences, College of Natural Sciences, Kangwon National University, Chuncheon 24341, Republic of Korea; 2 Hugenebio Institute, Bio-Innovation Park, Erom, Inc., Chuncheon 24427, Republic of Korea; 3 Biomedical Knowledge Engineering Laboratory, School of Dentistry and Dental Research Institute, Seoul National University, Seoul 08826, Republic of Korea; 4 AI Institute, Alopax-Algo, Co., Ltd., Seoul 06978, Republic of Korea; 5 Medical AI Team, Jonathan Wellcare Division, Acryl, Inc., Seoul 06069, Republic of Korea; 6 Integrated Medicine Institute, Loving Care Hospital, Seongnam 463400, Republic of Korea

**Keywords:** non-small-cell lung cancer, deep learning, graph convolutional network, cancer prediction, biomarker, gene ontology enrichment

## Abstract

Early diagnosis of lung cancer to increase the survival rate, which is currently at a low range of mid-30%, remains a critical need. Despite this, multi-omics data have rarely been applied to non-small-cell lung cancer (NSCLC) diagnosis. We developed a multi-omics data-affinitive artificial intelligence algorithm based on the graph convolutional network that integrates mRNA expression, DNA methylation, and DNA sequencing data. This NSCLC prediction model achieved a 93.7% macro F1-score, indicating that values for false positives and negatives were substantially low, which is desirable for accurate classification. Gene ontology enrichment and pathway analysis of features revealed that two major subtypes of NSCLC, lung adenocarcinoma and lung squamous cell carcinoma, have both specific and common GO biological processes. Numerous biomarkers (i.e., microRNA, long non-coding RNA, differentially methylated regions) were newly identified, whereas some biomarkers were consistent with previous findings in NSCLC (e.g., *SPRR1B*). Thus, using multi-omics data integration, we developed a promising cancer prediction algorithm.

## 1. Introduction

Lung cancer is the leading cause of cancer mortality, with the second-highest incidence rate worldwide as of 2020 [1]. Lung cancer has two major histological subtypes, non-small-cell lung cancer (NSCLC) and small-cell lung cancer. NSCLC, which accounts for approximately 85% of all lung cancers, can be further subdivided into lung adenocarcinoma (LUAD), lung squamous cell carcinoma (LUSC), and large cell carcinoma. At a drastically low level, lung cancer survival trends have shown some improvements from 26% among men with NSCLC at the time of diagnosis in 2001 to 35% among men diagnosed in 2014; this tendency is also true for women with NSCLC, and this improvement in NSCLC survival has also been confirmed for all races and ethnicities [2]. This observed progress in survival has taken advantage of targeted therapies and immunotherapies based on targetable or actionable gene mutations (e.g., *EGFR*, *BRAF*, and *KRAS*) and fusion/rearrangements (i.e., *ALK*, *ROS-1*, *RET*, *NTRK*) [3,4,5,6,7,8,9]. However, because a considerable number of patients are diagnosed at advanced stages and/or are negative for targetable gene alterations, there remains an immense clinical need for diagnosis as early as possible to increase the therapeutic response rate, low recurrence, and, ultimately, increase the survival rate. Aside from actionable mutations or structural variants, non-idiopathic, common diagnostic modalities are necessary for successful and effective early diagnosis of cancers.

Particularly in lung cancer, radiological images and histopathological phenotypes are more adequately considered as gold standards in tumor diagnosis and staging, but are also crucial predictors of response to therapies and prognosis. Further, artificial intelligence, primarily machine learning (ML) and deep learning (DL), has been successfully implemented in oncology in collaboration with radiology or histopathology to maximize sensitivity, specificity, and precision, especially in lung cancer [10,11,12]. In addition, combined with the ML/DL model, next-generation sequencing-based single omics, representing a part of the entire pool of genome, transcriptome, methylome, proteome, and metabolome, has gradually evolved not only to diagnose lung cancer but also guide therapeutic decision-making [13,14,15,16,17]. Numerous efforts have been made to develop the ML/DL algorithm, providing decision support tools that assist clinicians to correctly diagnose cancers. However, the implementation of classical diagnostic methods and single omics still has not resulted in sufficient resolution to prove causal relationships among genotypes, molecular aberrations, and etiological phenotypes. Each set of omics data may present its own key features associated with aspects such as transcription, post-translational modification, metabolic pathways, gene–environment interactions, the immune system, and the tumor microenvironment. Therefore, if integrated adequately, multi-omics analysis is reasonably expected to provide a more comprehensive view on cancer biomarker signatures and untangle the complexity of cancer initiation, progression, and metastasis in a synergistic manner.

Among such attempts, graph convolutional network (GCN)-based models have proven useful in multi-omics data stratification, correlation learning, weighted value order, and biomarker feature identification [18,19]. Thus, in this study, by applying the directed acyclic graph structure into the GCN architecture, we successfully integrated multi-modal features into a cancer prediction model. We primarily focused on the integration of omics features derived from RNA sequencing and DNA methylation sequencing. Subsequently, we evaluated the lung cancer prediction performance of the proposed model by means of sensitivity, specificity, accuracy, precision, area under the curve–receiver operating characteristic (AUC-ROC), macro F1-score, and weighted F1-score in comparison with those of other ML/DL models.

Implementing a screening program to diagnose patients at an early stage is a critical factor to decrease lung cancer-related deaths and improve survival. This multimodal model may provide a solid basis for early diagnosis of NSCLC.

## 2. Materials and Methods

### 2.1. Dataset

The lung cancer patient data comprised DNA sequencing, gene expression, and DNA methylation (Table 1). Baseline demographic data were retrieved and anonymized (Table 2). 

The Gene Expression Omnibus (GEO) database is used as an authorized array- and sequence-based database in cancer-related studies. Gene expression and DNA methylation data were retrieved to build the study dataset from June to August 2021.

The RNA-sequencing data (including mRNA, microRNA, and long non-coding RNA) of lung cancer were obtained from The Cancer Genome Atlas (TCGA) data repository in June–August 2021. RNA-sequencing data of patients with LUAD derived from TCGA contained 504 samples: stage I (n = 274), stage II (n = 120), stage III (n = 84), and stage IV (n = 26). Patients with LUSC were classified as stage I (n = 242), stage II (n = 160), stage III (n = 84), and stage IV (n = 7).

DNA methylation data of LUAD retrieved from the GDC Portal (https://portal.gdc.cancer.gov (accessed on 14 May 2021) included 502 patients: stage I (n = 279), stage II (n = 120), stage III (n = 81), and stage IV (n = 22). For LUSC patients, the data comprised stage I (n = 199), stage II (n = 142), stage III (n = 62), stage IV (n = 5). Genomic data, including single nucleotide variants (SNV), indels, fusions, and rearrangements, from 818 patients with NSCLC were obtained from the same GDC portal. GTEx was developed in an ongoing effort to establish a comprehensive public resource for studying tissue-specific gene expression and regulation. In this study, 578 lung samples from nondiseased GTEx v8 tissues were used.

Somatic mutations, single nucleotide polymorphisms, and gene expression data utilized in this study were mapped to protein-coding regions to make it easier to perceive the results and integrate all omics data.

### 2.2. Mathematical Concepts

Considering message passing as information flow, the aim of our main model is to build a function of data features on a subgraph of a multimodal data graph G=(V, E), with node set V and edge set E, which assists binary classifications with respect to each single-omics data item. To train this function, each GCN module in the model utilizes two types of matrices: the weight matrix W induced from each single-omics data item and the relation network among nodes in the subgraph. However, to produce matrices of two different types, one GCN module requires only one single-omics data item, not two inputs. To input single-omics data into the module, we represent it as a feature matrix $X\in {\mathbb{R}}^{n\times p}$, where n is the number of samples, and p is the number of input features. We set n differently with respect to the size of each dataset, and p differently with respect to the characteristics of the data, as described in Section 3.1.

In this module, an adjacency matrix $A\in {\mathbb{R}}^{n\times n}$ is formed with respect to each X. In this study, we set A such that $A_{ij}=sim\left(x_{i},\;x_{j}\right){\tilde{\delta }}_{ij}$ between nodes $v_{i}$ and node $v_{j}$, where $x_{i}$, $x_{j}\;$ are the rows of X, sim( · , · ) is the cosine similarity, and ${\tilde{\delta }}_{ij}=1$ if i≠j and 0 otherwise. Furthermore, we set sim( · , · )=0 if sim( · , · )<τ for some threshold τ, which was determined empirically. From this point forward, we focus on the mathematical structure of our model.

An original GCN can be constructed by multiple graph convolutional layers,

(1) $$H^{\left(d+1\right)}=f\left(H^{\left(d\right)},A\right)=\sigma \left(AH^{\left(d\right)}W^{\left(d\right)}\right),$$

where $H^{\left(d\right)}$ is the input of the d-th layer such that $H^{\left(0\right)}=X$, and $W^{\left(d\right)}$ is the weight matrix of the d-th layer. Here, σ(·) denotes an activation function, for which we choose LeakyReLU with a slope of 0.25 for the negative x-axis. Using the Kipf–Welling method [20], we can change A to the rearranged adjacency matrix $\hat{A}$, which is expressed as follows:

(2) $$\hat{A}={\tilde{D}}^{-\frac{1}{2}}\tilde{A}{\tilde{D}}^{-\frac{1}{2}},$$

where $\tilde{A}=A+I_{n}$ with the identity matrix $I_{n}$, and $\tilde{D}$ is a degree matrix such that ${\tilde{D}}_{ii}=\underset{j}{\sum }{\tilde{A}}_{ij}$. We take $\hat{A}$ as an adjacency matrix for each of our GCN modules.

Using the l-th single-omics data matrix $X^{l}\;\left(l=1,\;2\right)$ and $X^{l}$-optimized GCN module $GCN^{l}\left(\cdot \right)$, we can obtain the predicted label matrix ${\hat{Y}}^{l}=GCN\left(X^{l}\right)$. Here, we denote ${\hat{y}}^{l}{}_{k}$ to the k-th row of ${\hat{Y}}^{l}$. For the common true label matrix Y and its k-th row $y_{k}$, the cross-entropy loss function on the GCN–fully connected (FC) module can be written as

(3) $$L_{GCN}^{l}=\overset{n}{\underset{k=1}{\sum }}CE(y_{k},\;\;{\hat{y}}^{l}{}_{k})$$

where CE( · , · ) denotes the cross-entropy function. Similarly, in the terminal FC layer, namely the Mutation FC layer, the cross-entropy loss can be evaluated as follows:

(4) $$L_{F}=\overset{n}{\underset{k=1}{\sum }}CE(y_{k},\;\;F\left({\hat{y}}^{1}{}_{k},\;{\hat{y}}^{2}{}_{k}\right)),$$

where F( · , · ) denotes the layer operation on the Mutation FC layer, which includes two single-omics feature concatenations and rule-based filtering to match most suitable anti-cancer agent derived from DNA sequencing data. In brief, the total loss function L of our model is as follows:

(5) $$L=\alpha_{l}\overset{2}{\underset{l=1}{\sum }}L_{GCN}^{l}+\lambda L_{F}$$

with $\alpha_{l},\;\lambda \in \mathbb{R}$. Empirically, we set $\forall \alpha_{l}=1$ for the stability of the model.

### 2.3. DL Model Architecture and Weight Optimization

**Figure 1:**
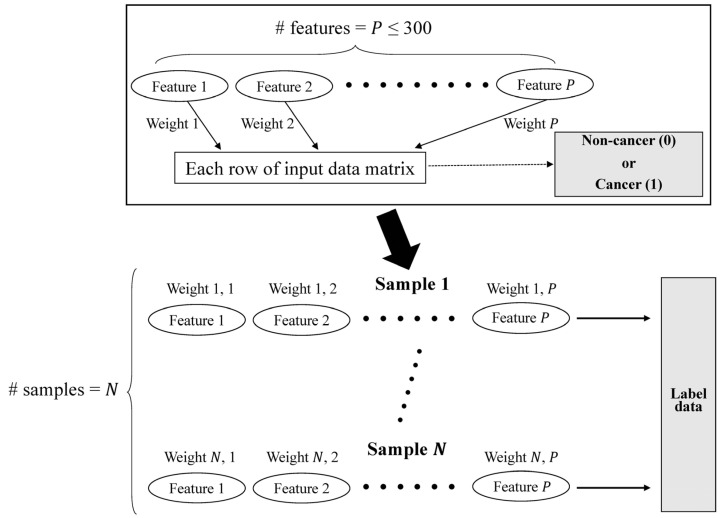
Data schema characterized by directed acyclic graph (DAG) structure. The DAG architecture is implemented to label the multi-omics data. This data-label flow is to avoid potential data duplication derived from graph-based preprocessing.

**Figure 2:**
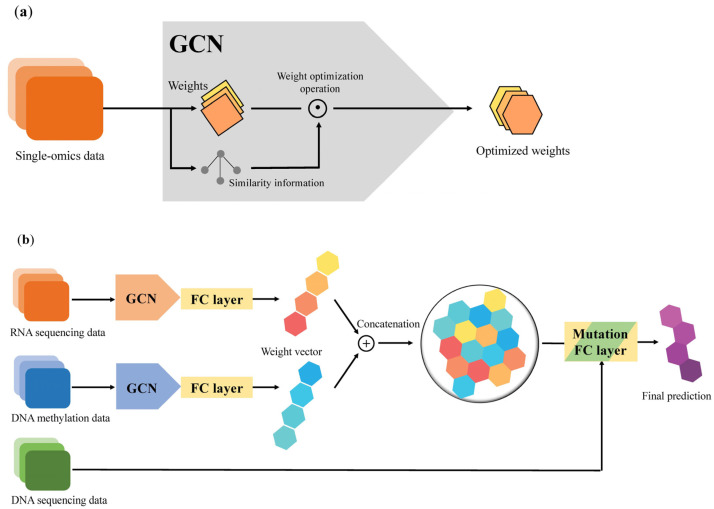
Overview of preprocessing module and graph convolutional network (GCN)-based non-small-cell lung cancer (NSCLC) prediction deep learning model. (**a**) GCN-based preprocessing module for weight optimization. (**b**) GCN-based NSCLC prediction algorithm. DNA sequencing data including targetable gene aberrations are served as discriminating predictors to match the most suitable therapeutic agents in the Mutation FC layer.

The preprocessing module was implemented by virtue of the GCN architecture, which is sufficient to emphasize the directed acyclic graph (DAG) structure. In accordance with the DAG structure, which represents the data flow from one activity to another and has a defined direction, we can smoothly parallelize the evaluation with each set of multi-omics data and can be free of the total number of features (Figure 1). Preprocessing equipped with a DAG structure can benefit from a decrease in the time required for training a model standardization of input forms of multi-omics data, which have different numbers of features (Figure 2a).

After applying a weighted sample similarity computed from the preprocessing module, each modified weight from the single-omics data is concatenated to an FC layer, and these vectors are then integrated as a multimodal weight vector in the same layer. In the Mutation FC layer, it collects all multimodal weight vectors and employs those vectors to generate the prediction of a binary label. Consequently, an NSCLC prediction model with a GCN–FC–FC architecture was established using multiple modalities (Figure 2b).

We selected the cosine function as a similarity function and Leaky ReLU with a slope of 0.25 for the negative x-axis as an activation function. We employed the Adam optimizer and cross-entropy loss, and Xavier weight initialization. We also pretrained our model for 500 epochs and then fine-tuned it for 2500 epochs. The number of hidden units in our model was 400 and the dimension of all FC layers was 200. The search space for all three learning rates (pre-training, training, and terminal classification) was {1 × 10^−7^, 5 × 10^−7^, 1 × 10^−6^, 5 × 10^−6^, 1 × 10^−5^, 5 × 10^−5^, 1 × 10^−4^, 5 × 10^−4^, 0.001, 0.005}. We selected a dropout rate of 0.5 and a sum method for node neighbor aggregations. The model was implemented using Python 3.8.10 and PyTorch 1.8.1 on an Intel^(R)^ Core^(TM)^ CPU i7-9800X, with 126 GB of memory, and a TITAN RTX 24 GB GPU with CUDA 10.2.

To optimize the weights of this GCN-based model, weight vectors of samples were obtained from the feature data of each sample, whose components were the feature values of the samples. Furthermore, we evaluated the similarities between two nodes in each sample–sample graph, especially within the 1-hop network of the graphs. With these similarities, the preprocessing module can restrict protruded components of the weight vector by adjusting the marginal value from the mean value of each feature, and hence smooth links, or directed edges, from each node to a binary label. For example, if one node with label 0 is located among nodes, and most nodes have label 1, then the weight of the link to label 0 decreases, and that of the link to label 1 increases at the same time. In this process, the similarity threshold τ presented in Section 2.2 is determined as a function with respect to the numbers of linked nodes.

### 2.4. Feature Identification

To find the most relevant features while avoiding redundant ones, log_2_ fold change and *p* value criteria were adopted for the selection of features or genes that were hypermethylated or hypomethylated and up- or down-regulated, respectively.

More specifically, after filtering for biomarkers with *p* value < 0.05 and absolute value of log_2_ fold change >0.5, thousands of features or genes were initially identified. To curtail the total number of biomarker candidates, we used Welch’s *t*-test for every feature or gene, on the assumption that the probability distribution is independent, displaying normality, and having unequal variances, according to the cancer positivity and negativity of the samples. The data were randomly divided into training, validation, and testing sets in 55%, 15%, and 30% ratios, respectively. The training set was utilized to establish the DL model, the validation set was used to determine the parameters of the model, and the testing set was applied to evaluate the performance of the DL algorithm. Five-fold cross-validation was performed to determine the optimal parameters for each training and validation model. We conducted the influence test for features by observing the increment of the F1-score between the inclusion and exclusion of each differentially methylated and expressed genes. Among the features, the feature for n-th gene is called Gn, and the degree of influence of Gn on the F1-score is defined as difference between the F1-score including Gn and the F1-score excluding Gn. The influence on the F1-score was calculated for all features, and features with high influence were selected according to influence on the F1-score ranking. The top 200 gene expression and 300 methylation features ranked by the GCN-based algorithm were selected, followed by subsequent analysis (Table S1). The diagnostic potentials of the model were determined and assessed using sensitivity, specificity, precision, accuracy, ROC curve, macro F1-score, and weighted F1-score.

### 2.5. Algorithm Comparisons

To compare our model with common ML/DL models, we implemented convolutional neural network (CNN), logistic regression, and naïve Bayes models, and set these models as the baseline. Although the original Naïve Bayes is an ML model, we built this model as a DL-structured model. We used an activation function, optimizer, and loss function for all baseline models, similar to our model, namely, Leaky ReLU, Adam, and cross-entropy loss. Moreover, we set the number of baseline hidden units to 400 and the dimension of the baseline FC layer to 200, based on the construction of our model. Furthermore, we attached baselines with hidden FC layers, whose numbers were the same as the hidden layers in our model.

For the CNN model, we chose a one-dimensional filter for convolution layers, max pooling with a kernel size of two, stride of one, and zero padding as the pooling method. Since we obtained better metric results when the dropout method was used, we used it at a rate of 0.5, instead of batch normalization. For the logistic regression model, we applied the batch normalizations for each batch and used a dropout method at a rate of 0.5. For the naïve Bayes model, we implemented each layer in a manner similar to the FC layer, using a standard normal distribution as a prior probability distribution.

The three baseline algorithms were compared for their prediction performance on this study’s NSCLC multimodal data. Seven metrics were adopted to compute the classification performances of the competitor methods. By applying the same preprocessed data, the performances of the implemented algorithms were compared pairwise via five-fold cross-validation.

### 2.6. Functional Annotation and Pathway Analysis

To further investigate the etiologic and molecular biological characteristics of NSCLC biomarkers revealed by this study, we conducted enrichment and pathway analysis implementing Gene Ontology (GO), Kyoto Encyclopedia of Genes and Genomes (KEGG), and REACTOME, which were performed using DAVID 6.8 or analyzed using Cytoscape 3.9.0. GO terms and pathways derived from enrichment were visualized using the ClueGO v2.5.8 and CluePedia v1.5.8 plug-ins. The enrichment library used for GO Biological Process terms is “GO_BiologicalProcess-EBI-UniProt-GOA-ACAP-ARAP_13.05.2021_00h00.” GO selection criteria for representative pathways included three–eight GO levels, a minimum of three genes/term, and mapped genes representing at least 4% of the total associated genes. The Kappa score was used to define term–term interactions and associate terms and pathways into functional groups based on the shared genes between the terms. Ensemble gene ID was used as a gene identifier for all analyses. FDR and *p* values corrected with Bonferroni less than 0.05 were considered significant. These analyses were performed and accessed between November and December 2021.

### 2.7. Statistics

All statistical data were confirmed using SPSS Statistics (version 22.0; IBM, Armonk, NY, USA) and MedCalc Statistical Software version 14.8.1 (MedCalc Software Ltd., Ostend, Belgium). Model performance comparisons were visualized using GraphPad Prism version 8.0.1 (GraphPad Software, San Diego, CA, USA). ROC curve analysis was performed to measure the area under the ROC curve (AUC), sensitivity, specificity, accuracy, and precision.

## 3. Results

### 3.1. Multi-Omics Datasets, Preprocessing, and Model Training

In the algorithm training step, we used the TCGA-LUAD, TCGA-LUSC, and Korean lung carcinoma datasets (GSE40419). These sets contain three types of data, including mRNA gene expression, DNA methylation, and genomic data, which are suitable for disease classification. We primarily focused on transcriptomics and DNA methylation in the identification of lung cancer prediction signatures. Genomic data, including SNVs, indels, and fusions/rearrangements, are expected to discriminate between the predictors that match the most suitable chemo- and/or immune-therapeutic agents.

The datasets were split into 777 training datasets (55.7%), 195 validation datasets (14.0%), and 423 test datasets (30.3%). These data included more than 10,000 features, which may affect the classification performance and undermine the algorithm training. To remove noise and redundant features from each multi-omics dataset, we first employed Welch’s *t*-test (*p* < 0.05) on reliable assumptions. Then, we employed the influence test for features by observing the increment of the F1-score between the inclusion and exclusion of each feature. Using these approaches, we were able to specify 200 RNA-sequencing and 300 methylation features.

We used the macro F1-score, weighted F1-score, and accuracy as performance metrics, and the AUC-ROC score as sub-metrics. For efficient training and validation, we used five-fold cross-validation. Training was performed to carry out initial class prediction using omics features and the corresponding sample similarity generated from omics data.

Our GCN-based algorithm with DAG structure can diagnose lung cancer, more specifically NSCLC, based on two single omics data, transcriptomics, and DNA methylation.

Major RNA expression and DNA methylation features identified by this GCN-based prediction algorithm were parallel to the GO enrichment and pathway analysis (Table 3).

### 3.2. NSCLC Prediction Model Validation

Regarding accuracy and specificity, five-fold cross-validation of this NSCLC prediction model (Figure S1) showed that the performance of DNA methylation single-omics data was slightly better than that of RNA-sequencing unimodal data. Notably, in the case of specificity, the metric values were recorded as 1.0, in most folds, after rounding-off. This case implies that the influence of methylation data is exiguously significant, where our model correctly predicts a healthy subject as non-cancerous. Conversely, we observed that the F1-scores using each data point are almost the same on average. Because the F1-score is a discrimination score for both non-cancer/cancer cases of NSCLC, the observation clarifies that the effect scales of both data are nearly equal in the simultaneous classification of both cases.

### 3.3. Comparison of NSCLC Prediction Model with Other Classifier Models

**Figure 3:**
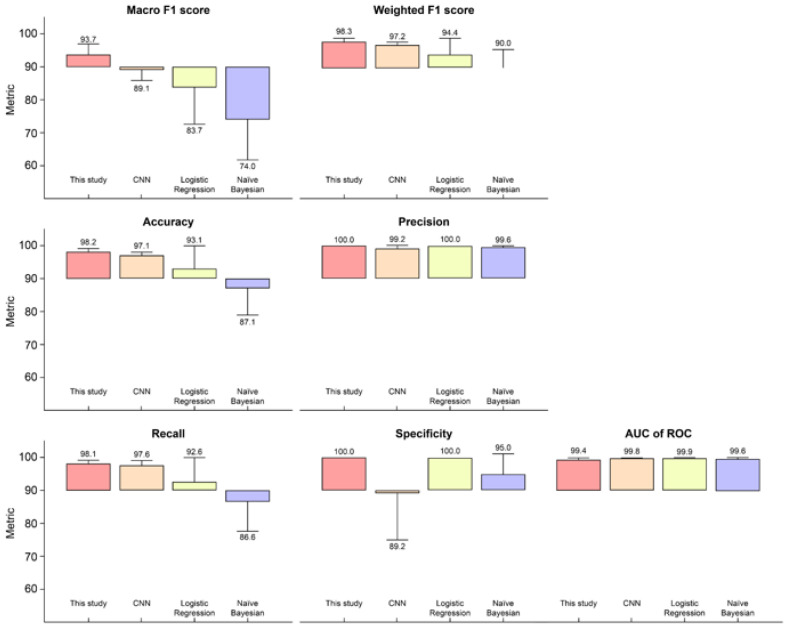
Performance comparisons of NSCLC prediction model with alternative classifier models. Pairwise comparisons of the implemented algorithm performances were analyzed via five-fold cross-validation. To improve discrimination, the metric cut-off was set at 90%. The standard deviation of each performance is illustrated by a vertical error bar. AUC of ROC denotes area under the receiver operating characteristic curve.

The prediction performance of this model was compared with that of existing DL and ML algorithms: CNN, logistic regression, and naïve Bayes. All four methods were compared using the same preprocessed data and the mean ± standard deviation of the evaluation metrics (Figure 3 and Table S3). Actual prediction results of this model based on TCGA samples were compared with those of other classifier models (Table S4).

To compare the model performances of the baseline and the proposed model, we adopted sensitivity, specificity, accuracy, precision, AUC-ROC, macro F1-score, and weighted F1-score for the binary classification of NSCLC. The best model varied depending on the performance metric, but the proposed model was at least equal to or higher than that of the other multi-omics integrative algorithms on six of the seven metrics we analyzed. In other words, the proposed model outperformed the other algorithms on most performance metrics.

More specifically, when comparing the models with the same number of hidden layers and the same activation function, we observed that our model outperformed the competitors in terms of accuracy, *sensitivity*, *macro F*1-*score*, and *weighted F*1*-score*. *Precision*, which is the ratio of correctly labeled samples to all labeled samples, can vary depending on the classifier threshold. Meanwhile, the *sensitivity* may remain unchanged.

(6) $$Precision=\frac{True\;positives}{True\;positives+False\;positives}\;$$

(7) $$Sensitivity=\frac{True\;positives}{True\;positives+False\;negatives}$$

where *true positives* indicate the number of NSCLC samples that have been correctly predicted, *false positives* denote the number of non-cancer samples that are misclassified as cancer samples, and *false negatives* signify the number of NSCLC samples that are misclassified as non-cancer samples. The *F*1*-score*, the harmonic mean of *precision* and *sensitivity*, is a useful measure in binary classification and was calculated according to Equation (8):

(8) $$F1\;score=\frac{2\times Precision\times Sensitivity}{Precision+Sensitivity}\;$$

*Macro F*1 can be computed using the sum of the *F*1*-score* for *actual positives* and *actual negatives*, and was calculated according to Equation (9):

(9) $$Macro\;F1=\frac{F1\;of\;actual\;positives+F1\;of\;actual\;negatives}{2}\;$$

Our NSCLC prediction model achieved a 93.7% *macro F*1*-score*. The highest *macro F*1*-score* indicated that the values for *false positives* and *false negatives* were sufficiently low to prove that this algorithm is best for classifying the correct class (Figure 3).

To calculate the metrics for each label and adjust their *macro F*1 weighted by both the *actual positives* and *negatives*, *weighted F*1 was applied as per Equation (10):

(10) $$Weighted\;F1=\frac{\left(No.\;of\;actual\;positives\times F1\;of\;actual\;positives\right)+\left(No.\;of\;actual\;negatives\times F1\;of\;actual\;negatives\right)}{2}\;$$

*Weighted F*1, considering the number of actual occurrences per label, was 98.3% and it outperformed the competitor model, with an imbalanced dataset, that is, DNA methylation in this study. The proposed NSCLC prediction model demonstrated that it outperforms three other models applied using identical frameworks that are desirable for multi-omics data classification capability on identical datasets.

### 3.4. Functional Annotation and Pathway Analysis

**Figure 4:**
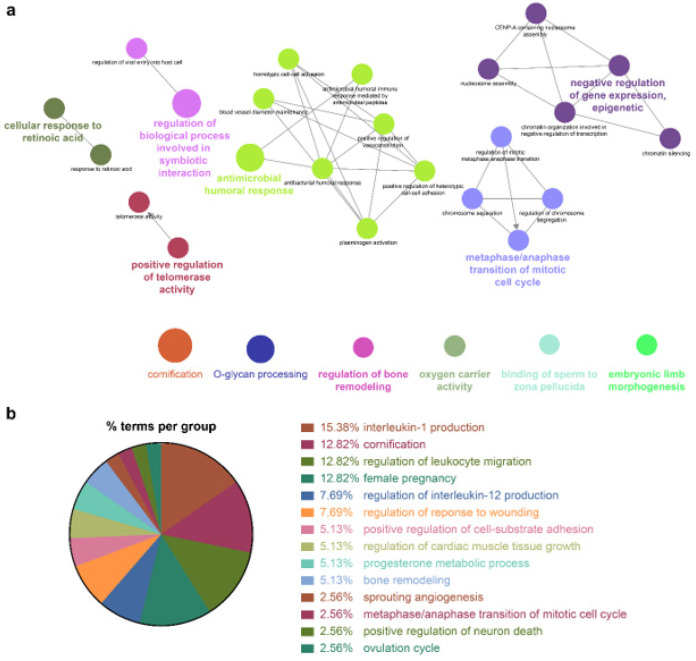
GO enrichment and pathway analysis of NSCLC features. (**a**) Visualized networks of enriched GO “Biological Process” terms of NSCLC were grouped based on shared genes (Kappa score threshold = 0.4). Enriched terms by *p* value corrected with Bonferroni were retained as the functional description. The node size is proportional to the degree of significance. (**b**) % terms per group represents the proportion of GO terms in the NSCLC features.

**Figure 5:**
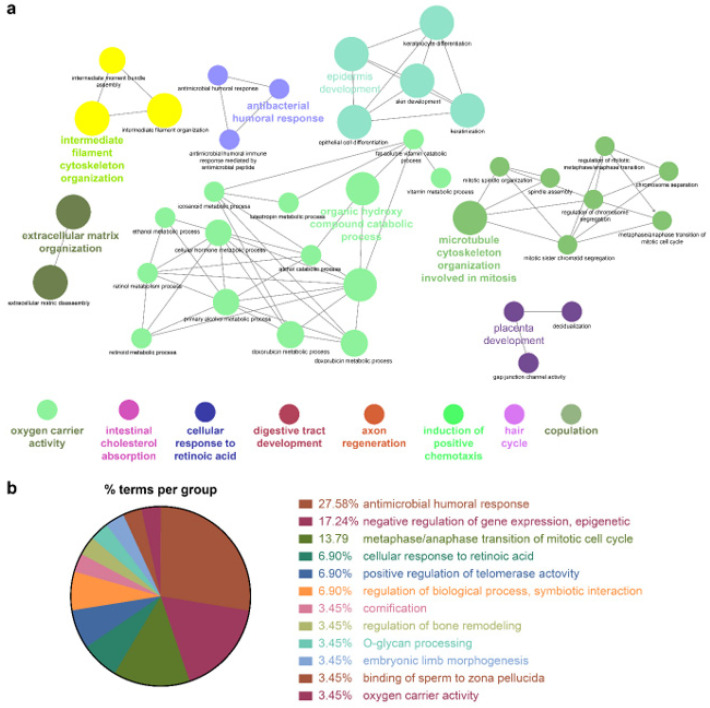
GO enrichment and pathway analysis of LUAD features. (**a**) Enriched GO “Biological Process” terms of LUAD were grouped based on shared genes (Kappa score threshold = 0.4). Enriched terms by *p* value corrected with Bonferroni were retained as the functional description. The node size is proportional to the degree of significance. (**b**) % terms per group represents the proportion of GO terms in the LUAD features.

**Figure 6:**
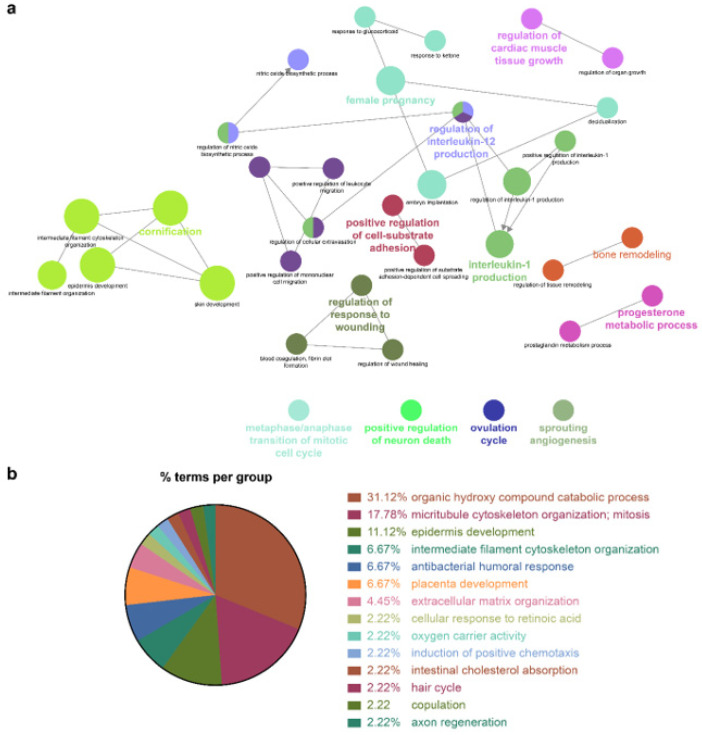
GO enrichment and pathway analysis of LUSC features. (**a**) Enriched GO “Biological Process” terms of LUSC were grouped based on shared genes (Kappa score threshold = 0.4). Enriched terms by *p* value corrected with Bonferroni were retained as the functional description. The node size is proportional to the degree of significance. (**b**) % terms per group represents the proportion of GO terms in the LUSC features.

Because the biomarkers identified by the NSCLC prediction model were quite diverse, further analysis of their molecular functions and biological processes was performed. LUAD and LUSC not only shared identical biological processes but also exhibited specific processes. GO term enrichment, KEGG, and REACTOME pathway analyses were performed on the identified biomarkers using Cytoscape software 3.9.0. and the DAVID database. In the NSCLC multimodal features, the GO term of cornification (GO:0070268) originated from LUAD features, whereas epidermis development (GO:0008544) was derived from LUSC features (Table 4, Figure 4, Figure 5 and Figure 6).

Genes associated with the two GO terms cornification and epidermis development showed distinct downregulation in NSCLC (Figure S2). GO terms related to the inflammatory response, that is, positive regulation of IL-1 production (GO:0032732), regulation of IL-12 production (GO:0032655), and the IL-17 signaling pathway (GO:04657), were enriched in NSCLC. In this inflammatory response network, up- and downregulation were intermingled, but upregulated genes prevailed (Figure S2 and Table 4).

Cellular responses to retinoic acid (GO:0071300) and antimicrobial humoral responses (GO:0019730) were shared by both LUAD and LUSC (Figure 5 and Figure 6 and Table 4). REACTOME pathway analysis indicated that the formation of the cornified envelope (R-HAS:6809371) pathway also had a common term in both LUAD and LUSC (Table 4). O-linked glycosylation (R-HAS:5173105) was distinctive in LUAD, and degradation of the extracellular matrix (R-HAS:1474228) was peculiar to LUSC (Table 4). KEGG pathway analysis revealed that the REACTOME term estrogen signaling pathway (KEGG:04915) was highly enriched in LUSC features, which was in turn applied to the NSCLC multimodal model. In this estrogen signaling pathway, *KRT13*, *KRT14*, *KRT15*, *KRT16*, and *KRT17* were enriched genes, which allowed us to predict that these genes contribute to the downregulation of the estrogen signaling pathway (Table 4).

All the details of the GO terms, KEGG, and REACTOME pathway analyses are displayed in Table 4 and Table S2. GO enrichment analysis using Cytoscape and its plug-ins ClueGO and CluePedia was successfully reproduced in the analysis using the DAVID database. Representative enriched GO terms (-Log_10_ *p* value <2.4; fold change +5–−8) of NSCLC multimodal features, analyzed from the DAVID database, are summarized in Figure S3.

## 4. Discussion

Owing to the exponential growth of omics technologies and NGS-based big consortium studies, labeled omics datasets with comprehensive annotations are continuously being developed. Therefore, it is increasingly important to employ labeled omics data to highly accurately predict disease diagnosis, tumor grading, and subtyping. Considering approximately 85% of lung cancers, NSCLC has two of the most common subtypes, LUAD and LUSC, which have considerably different biological signatures, although they are usually treated in the same way and classified identically into NSCLC. There are few biomarkers of NSCLC based on the implementation of multimodal feature selection methods. One of the main reasons for this is histologic and molecular heterogeneity, even within the same histological subtype. Hence, to accurately and robustly predict NSCLC, this study focused on the multi-omics integrative classification of NSCLC.

Although multi-omics data integration usually leads to better outcomes, some studies have suggested that this is not always the case. Existing reviews comprehensively cover this challenging issue [21,22]. Poor performance can arise if the algorithm is not adapted for a particular aim or for particular multi-omics datasets. Some ML/DL models cannot handle enormous matrices, noise, and outliers, which are worse in multi-omics studies. However, as discussed by Picard et al., if an algorithm and integration strategy are chosen discretely, multi-omics-based models should always outperform single-omics models [21].

A supervised omics integrative model has been reported to take advantage of a GCN for multi-omics data learning to perform effective disease classification and biomarker identification. Wang et al. utilized both omics features and correlations among samples represented by similarity networks for better classification capability in cancers and Alzheimer’s disease [19]. Ramirez et al. reported that a GCN with prior knowledge in the form of protein–protein interaction networks and gene co-expression networks was able to achieve excellent prediction accuracies (89.9–94.7%) among 33 cancer types and normal tissue on TCGA data [23].

Numerous multi-omics integration studies have proposed the applicability of DL for cancer diagnosis and prognosis. However, there are a few challenges and limitations to the extensive implementation of multi-omics DL in clinical practice. First, the fundamental problem encountered is the difficulty in resolving the relationship between the compressed features and biological meanings. Therefore, to verify the explainable performance in real-world applications, a clinical study must be carried out with external cohorts, followed by complete functional analysis. Second, owing to the large number of parameters, DL algorithms are difficult to train, must be tuned accurately, have unclear methods concerning handling data variability derived from data transformation and normalization, and often experience overfitting. In addition, their performance depends heavily on the size of the samples, which is limited [21,24]. Strict performance evaluation is particularly demanding owing to the innate high complexity of artificial intelligence networks, as apparently well-performing DL models might use inadvertent and possibly false features and respond unexpectedly to seemingly irrelevant changes in input data. Failure to perform a strict evaluation might have diminished the credibility of the research findings and would be worthless in the clinical field [25].

Another issue likely to be encountered when incorporating ML models trained using multi-omics data is the expense. It might not be practical to perform multimodal sequencing or microarray analyses routinely for all new patients. To implement cost-effective and high-throughput analysis for NSCLC diagnosis and/or prognosis, we further differentiate central or core biomarkers (approximately 30–50) out of a few hundreds of multi-omics feature pools, while maintaining comparable performance in NSCLC prediction.

The superior performance of the macro F1 metric of this NSCLC prediction model can be explained by a previous DL cancer classification study. The macro F1-score has been applied as a useful measure of the effectiveness of individual algorithms on the rare classes because macro F1 is more heavily influenced by performance on the minority classes, that is, cancers vs. non-cancers [26]. There is a need for further evaluation of this NSCLC prediction performance by training and testing on different patient cohorts from different institutions and ethnicities.

Gene Ontology (GO) annotates genes (i.e., features) using ontology. GO analysis is widely used for specifying cellular location, molecular function, and biological process. One of main purpose of implementing GO is to perform enrichment analysis on a given feature list [27]. GO annotations smoothen the way for capturing the complexity of gene and function relationships. Pathway analysis (e.g., KEGG, STRING, PANTHER) groups genes or features into ‘pathways’ which basically enlist them to participate in the same biological process. Thus, it can identify the specific protein functions, biological pathways, and physical interactions that are enriched in a given feature panel [28]. This study unveiled genes that were more correlated with LUAD, or more specifically to LUSC, and universally contributed to both subtypes (Table 4). A particularly enriched GO term in this NSCLC algorithm is cornification (GO:0070268), which is a distinct type of cell death featuring terminal keratinocyte differentiation with a slow, coordinated process time spatially, allowing the formation of a dead cell (corneocyte) layer to create a physical barrier for the skin [29]. The SPRR family of proteins is located in the region of the epidermal differentiation complex. Wang et al. demonstrated that *SPRR1A* expression in LUAD indicates a more advanced stage and unfavorable prognosis [30]. Further, Zhang et al. reported that *SPRR1B* expression in LUAD may predict poor prognosis [31]. Additionally, Patterson et al. stated that the loss of *SPRR1B* expression disrupts or alters the cornification process, resulting in irreversible malignant transformation [32]. We intend to investigate the molecular functions of the SPRR family genes (Table 4) unveiled from this study and their correlation with NSCLC.

This study demonstrated that *KRT6A* (log_2_FC −5.47), *KRT14* (log_2_FC −3.71), *S100A2* (log_2_FC −3.23), and *KRT17* (log_2_FC −2.56) have significant diagnostic potential for NSCLC (Table S1), consistent with a previous study using overlapping feature selection methods in which these genes were ranked as having one of the highest diagnostic potentials for classifying LUAD and LUSC [33]. In alignment with top-ranked features, GO biological processes were similarly concatenated with “Epidermis development,” “Intermediate filament cytoskeleton organization,” and “Intermediate filament-based process.” These GO terms, originating from LUSC, suggested that LUSC expresses more genes related to epidermal development and cytoskeleton organization, which is different from LUAD.

Novel microRNA, long non-coding RNA, and differentially methylated regions, which regulate or are the nearest genes to these RNAs, were unveiled. The exact mechanisms underlying these novel RNA biomarkers in NSCLC development should be clarified in future studies.

## 5. Conclusions

In this study, we developed a novel NSCLC biomarker identification framework through multi-omics data integration using a GCN-based algorithm and demonstrated the high precision and accuracy of its binary classification (cancer vs. non-cancer). This algorithm effectively identified salient biomarkers from integrated multi-omics data, which exhibited superior performance. There are downsides and limitations regarding the implementation of multi-omics DL. This study demonstrated that employing the proposed model to handle multi-omics data can help eliminate them. The future direction of the research is to achieve a more applicable DL algorithm and multi-omics-based diagnostic device, prove its clinical utility in large-scale human clinical trials, and ultimately support the improvement of the NSCLC survival rate, which remains drastically low.

## Figures and Tables

**Table 1:** Dataset characteristics.

Data	RNA Sequencing	DNA Methylation	DNA Sequencing
Dataset accession ID	TCGA-LUAD, TCGA-LUSC, GSE40419, GTEx v8 Lung	TCGA-LUAD, TCGA-LUSC	TCGA-LUAD, TCGA-LUSC
Features	957	423	230
Cancer patients	1122	1117	818
Healthy subjects	763	28	22

**Table 2:** Baseline demographics of subjects.

Data	RNA Sequencing	NSCLC			Non-Cancer		
		TCGA, GSE40419		TCGA, GSE40419			GTEx
Subjects	n	1122		185			578
	Female, n (%)	451 (40.2)		81 (43.8)			183 (31.6)
Age	Mean (SD)	66.09 (9.44)		67.24 (9.87)			-
	≥50, n (%)	1033 (92.1)		174 (94.1)			412 (71.2)
Race/ethnicity	White, non-Hispanic (%)	737 (65.7)		97 (52.4)			-
	Black, non-Hispanic (%)	84 (7.5)		6 (3.2)			-
	Asian incl. Hawaiian or Pacific islander (%)	103 (9.2)		77 (41.6)			-
	Hispanic (%)	19 (1.7)		0 (0)			-
	Others (%)	179 (15.9)		5 (2.7)			-
Cancer stage	I (%)	592 (52.8)		-			-
	II (%)	298 (26.6)		-			-
	III (%)	181 (16.1)		-			-
	IV (%)	37 (3.3)		-			-
	Missing (%)	14 (1.2)		-			-
				
**Data**	**DNA Methylation**		**NSCLC**			**Non-Cancer**	
			
Subjects	n		843			74	
	Female, n (%)		350 (41.5)			28 (37.8)	
Age	Mean (SD)		66.09 (9.66)			66.86 (10.82)	
	≥50, n (%)		768 (91.1)			68 (91.8)	
Race/ethnicity	White, non-Hispanic (%)		624 (74)			58 (78.5)	
	Black, non-Hispanic (%)		76 (9)			6 (8.1)	
	Asian incl. Hawaiian or Pacific islander (%)		13 (1.6)			1 (1.3)	
	Hispanic (%)		16 (1.9)			2 (2.7)	
	Others (%)		114 (13.5)			7 (9.4)	
Cancer stage	I (%)		432 (51.2)			-	
	II (%)		250 (29.6)			-	
	III (%)		129 (15.3)			-	
	IV (%)		24 (2.9)			-	
	Missing (%)		8 (1.0)			-	

Data presented as n.

**Table 3:** Major RNA sequencing and DNA methylation features for NSCLC prediction.

Ensemble Gene ID		Gene Symbol		Log_2_ Fold Change		Chromosome		GRCh38, Start	GRCh38, End		Length		Strand	Gene Type
ENSG00000171564		FGB		8.861		4		154,563,011	154,572,807		9796		+	Protein coding
ENSG00000110680		CALCA		8.266		11		14,966,668	14,972,351		5683		-	Protein coding
ENSG00000164266		SPINK1		6.680		5		147,824,572	147,831,671		7099		-	Protein coding
ENSG00000169469		SPRR1B		5.858		1		153,031,203	153,032,900		1697		+	Protein coding
ENSG00000176153		GPX2		5.854		14		64,939,152	64,942,905		3753		-	Protein coding
ENSG00000167656		LY6D		5.698		8		142,784,882	142,786,539		1657		-	Protein coding
ENSG00000143320		CRABP2		5.559		1		156,699,606	156,705,816		6210		-	Protein coding
ENSG00000205420		KRT6A		5.473		12		52,487,176	52,493,257		6081		-	Protein coding
ENSG00000099953		MMP11		5.135		22		23,768,226	23,784,316		16,090		+	Protein coding
ENSG00000196611		MMP1		5.042		11		102,789,401	102,798,160		8759		-	Protein coding
ENSG00000204305		AGER		−5.001		6		32,180,968	32,184,322		3354		-	Protein coding
ENSG00000168484		SFTPC		−4.729		8		22,156,913	22,164,479		7566		+	Protein coding
ENSG00000165197		VEGFD		−3.585		X		15,345,596	15,384,413		38,817		-	Protein coding
ENSG00000164530		PI16		−3.539		6		36,948,263	36,964,837		16,574		+	Protein coding
ENSG00000133800		LYVE1		−3.304		11		10,556,966	10,611,689		54,723		-	Protein coding
								
**CpG site_ID**	**Abs. Diff. ^1^**		**Methyl. Pattern**		**Chromosome**		**UCSC_RefGene_Name**			**UCSC_RefGene_Group**		**UCSC_CpG_Islands_Name ^2^**		
cg25774643	0.561		Hypermethylation		11		SCT			TSS200		chr11:626728-628037		
cg03502002	0.464		Hypermethylation		18		GALR1;GALR1			1stExon;5′UTR		chr18:74961556-74963822		
cg22674699	0.532		Hypermethylation		2		HOXD9			1stExon		chr2:176986424-176988291		
cg18322569	0.495		Hypermethylation		1		BARHL2;BARHL2			5′UTR;1stExon		chr1:91182509-91182857		
cg19760241	0.501		Hypermethylation		17		LHX1			Body		chr17:35291899-35300875		
cg20399616	0.474		Hypermethylation		12		BCAT1			Body		chr12:25055599-25056246		
cg21472506	0.517		Hypermethylation		2		OTX1			3′UTR		chr2:63283936-63284147		
cg04415798	0.490		Hypermethylation		14		PAX9			5′UTR		chr14:37126786-37128274		
cg18077971	0.469		Hypermethylation		2		PAX3			TSS1500		chr2:223162946-223163912		
cg27071152	0.474		Hypermethylation		7		LOC646999			Body		chr7:39649253-39649510		
cg07860213	0.486		Hypermethylation		8		PRDM14			Body		chr8:70981873-70984888		
cg26799474	0.387		Hypomethylation		2		CASP8			5′UTR		Not applicable		
cg25247520	0.432		Hypomethylation		8		MIR1204;PVT1			TSS200;Body		chr8:128806081-128806899		
cg07551060	0.399		Hypomethylation		10		GRK5			Body		chr10:121075133-121075401		
cg06051311	0.404		Hypomethylation		6		TRIM15			5′UTR;1stExon		chr6:30130969-30131093		

^1^ Absolute differences in beta-value between cancer and non-cancer samples.^2^ CpG sites is based on the reference human genome (GRCh37/hg19) assembly.

**Table 4:** Major enriched GO, KEGG, and REACTOME terms in NSCLC and its subtype LUAD and LUSC.

	ID	Category	Term	Group *p* Value Corrected with Bonferroni	% Associated Genes ^1^	Nr. Genes ^2^
**NSCLC**	GO:0070268	GO biological process	Cornification	2.7 × 10^−6^	13.79	16
	GO:0008544	GO biological process	Epidermis development	2.7 × 10^−6^	4.03	20
	GO:0032732	GO biological process	Positive regulation of interleukin-1 production	1.1 × 10^−4^	6.25	5
	GO:0032655	GO biological process	Regulation of interleukin-12 production	2.8 × 10^−4^	8.06	5
	GO:0010811	GO biological process	Positive regulation of cell-substrate adhesion	1.6 × 10^−3^	5.22	7
	KEGG:04657	KEGG pathway	IL-17 signaling pathway	9.3 × 10^−4^	5.32	5
	KEGG:04915	KEGG pathway	Estrogen signaling pathway	1.1 × 10^−3^	5.07	7
	R-HSA:6809371	Reactome pathway	Formation of the cornified envelope	6.2 × 10^−9^	12.31	16
**LUAD**	GO:0070268	GO biological process	Cornification	2.4 × 10^-7^	8.62	10
	GO:0019730	GO biological process	Antimicrobial humoral response	7.0 × 10^−6^	4.73	7
	GO:0045814	GO biological process	Negative regulation of gene expression, epigenetic	1.2 × 10^−3^	4.84	6
	GO:0016266	GO biological process	O-glycan processing	1.3 × 10^−3^	7.58	5
	GO:0007091	GO biological process	Metaphase/anaphase transition of mitotic cell cycle	7.8 × 10^−3^	4.76	3
	GO:0071300	GO biological process	Cellular response to retinoic acid	9.4 × 10^−3^	4.17	3
	KEGG:04613	KEGG pathway	Neutrophil extracellular trap formation	5.5 × 10^−5^	5.26	10
	R-HSA:6809371	Reactome pathway	Formation of the cornified envelope	2.8 × 10^−6^	7.69	10
	R-HSA:5173105	Reactome pathway	O-linked glycosylation	1.4 × 10^−3^	5.41	6
**LUSC**	GO:0008544	GO biological process	Epidermis development	1.4 × 10^−18^	6.65	33
	GO:1901616	GO biological process	Organic hydroxy compound catabolic process	3.0 × 10^−7^	9.64	8
	GO:0030198	GO biological process	Extracellular matrix organization	2.2 × 10^−6^	4.04	18
	GO:0019730	GO biological process	Antimicrobial humoral response	3.9 × 10^−3^	4.73	7
	GO:0005344	GO biological process	Oxygen carrier activity	4.8 × 10^−3^	15.79	3
	GO:0071300	GO biological process	Cellular response to retinoic acid	3.0 × 10^−2^	4.17	3
	KEGG:04915	KEGG pathway	Estrogen signaling pathway	7.4 × 10^−3^	5.07	7
	R-HSA:6809371	Reactome pathway	Formation of the cornified envelope	5.9 × 10^−12^	15.38	20
	R-HSA:1474228	Reactome pathway	Degradation of the extracellualr matrix	4.2 × 10^−4^	7.86	11

^1^ % Associated Genes indicates percentage of genes found from the input gene lists. ^2^ Nr. Genes denotes number of genes.

## Data Availability

All data generated or analyzed during this study are included in this published article and its Supplementary Information files.

## Supplementary Materials

The following supporting information can be downloaded at: https://www.mdpi.com/article/10.3390/biom12121839/s1, Figure S1: Heatmap plot representing 5-fold cross-validation results; Figure S2: Cytoscape string network analysis of NSCLC RNA features; Figure S3: Fold enrichment of GO biological process terms in NSCLC; Table S1: Multimodal features of NSCLC; Table S2: GO enrichments in LUAD, LUSC, and NSCLC; Table S3: Performance comparisons of NSCLC prediction model with different feature selection methods; Table S4: Actual prediction results of TCGA samples and their confusion matrices.
