# Supplementary figures and images for: Deep-Learning Algorithm and Concomitant Biomarker Identification for NSCLC Prediction Using Multi-Omics Data Integration
Source: Biomolecules. 2022 Dec 8;12(12):1839. doi: 10.3390/biom12121839 (PMC9775093; doi:10.3390/biom12121839)

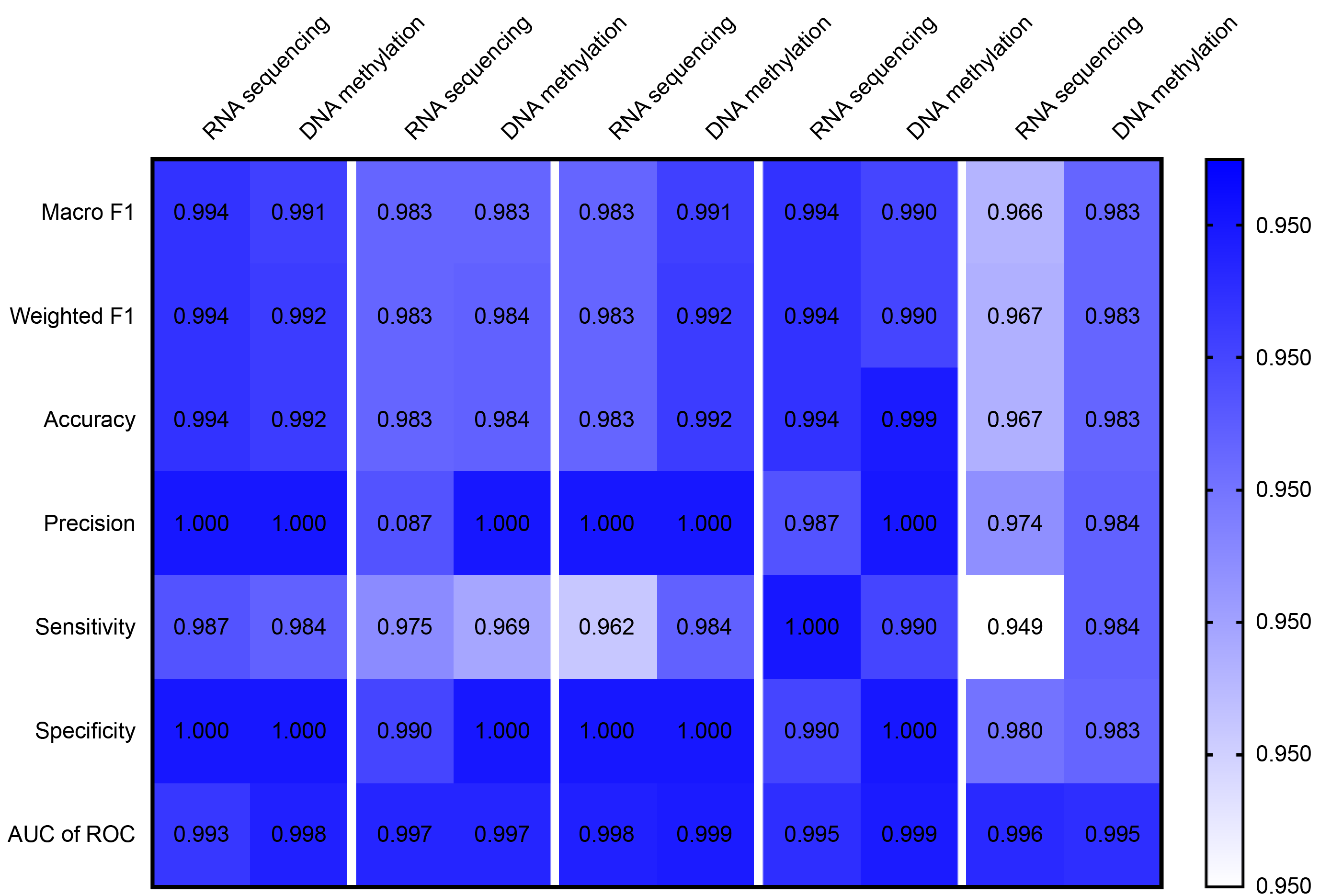

Supplement: Supplementary file 1 [file biomolecules-12-01839-s001.zip › Figure S1.tif]

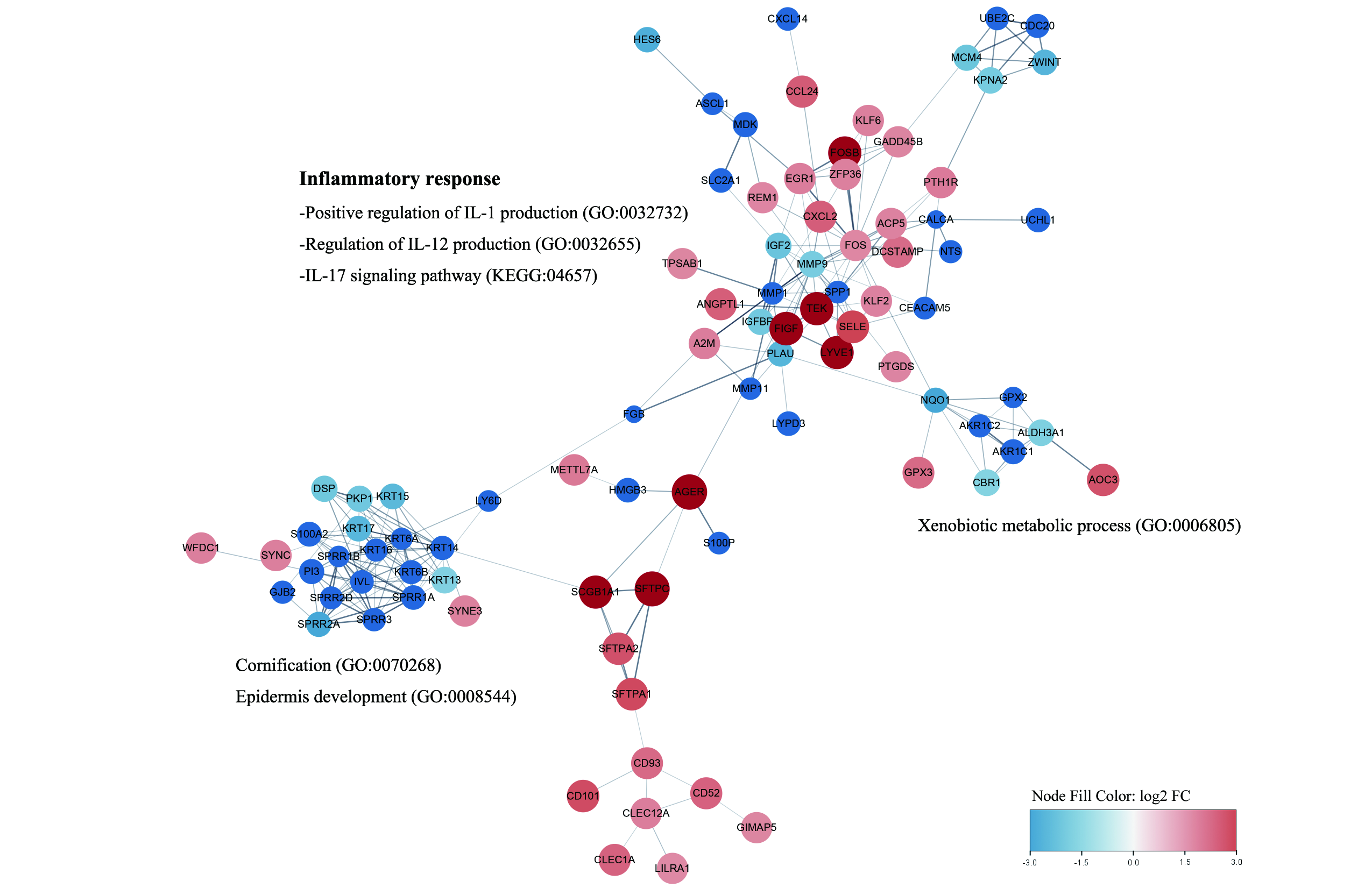

Supplement: Supplementary file 1 [file biomolecules-12-01839-s001.zip › Figure S2.tif]

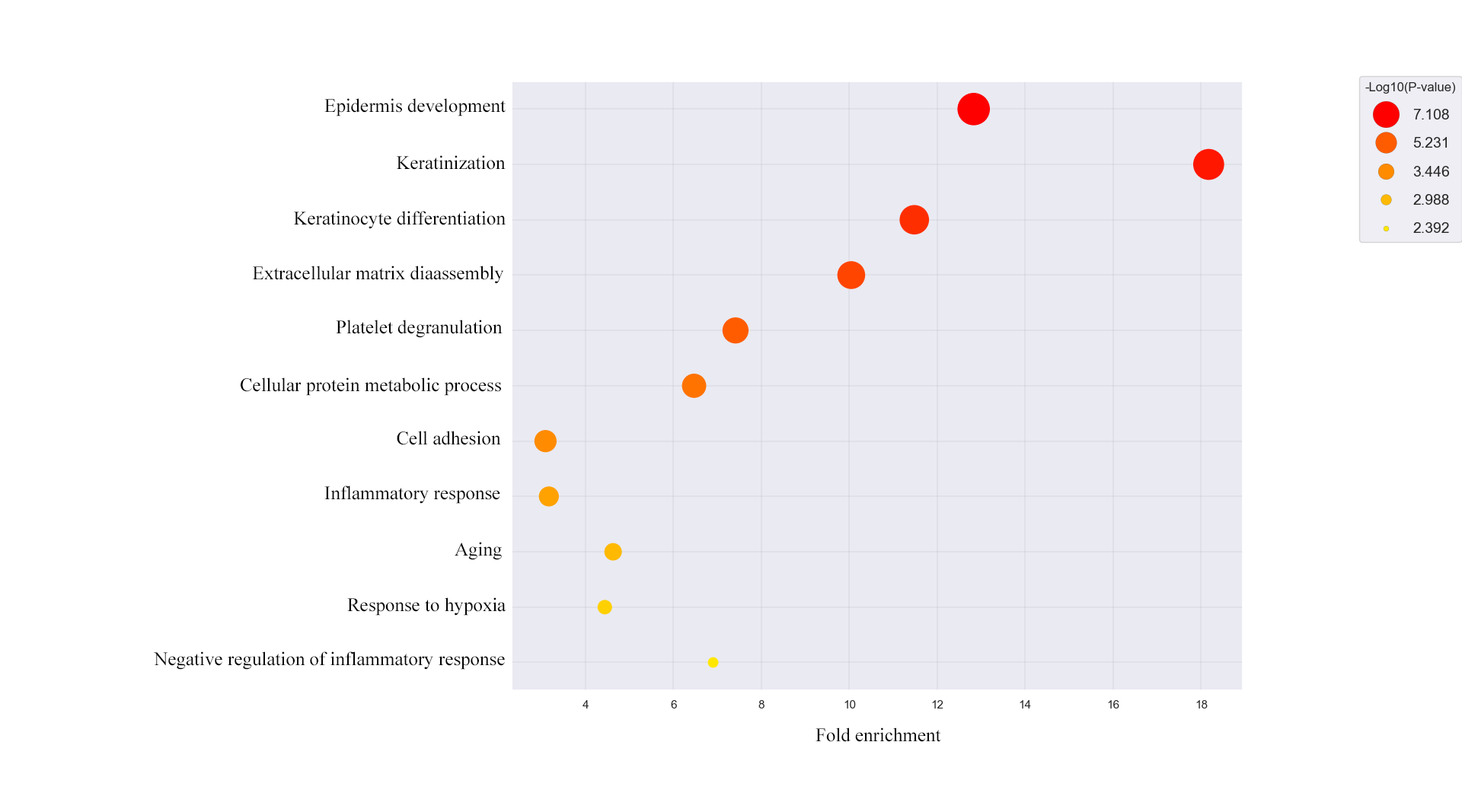

Supplement: Supplementary file 1 [file biomolecules-12-01839-s001.zip › Figure S3.tif]
